# Nutrition literacy and dietary self-management in breast and gynecologic cancer survivorship

**DOI:** 10.3389/fpubh.2026.1902901

**Published:** 2026-07-28

**Authors:** Shuai Teng, Fengfeng Teng, Yuguang Zhuang, Chenyan Zhao, Saisai Zhao, Yiqin Yao

**Affiliations:** 1 School of Education, Weifang University, Weifang, Shandong, China; 2 Changle Hetou Primary School, Weifang, Shandong, China; 3 Binzhou Development Zone No. 1 Middle School, Binzhou, Shandong, China; 4 Linyi No. 2 Experimental Primary School, Linyi, Shandong, China; 5 Hanting Experimental Primary School, Weifang, Shandong, China; 6 Fangzi Industrial Development Zone Experimental School, Weifang, Shandong, China

**Keywords:** breast cancer, cancer nutrition, dietary self-management, fear of recurrence, gynecologic cancers, nutrition literacy, psychological distress, survivorship care

## Abstract

Women living with and beyond breast cancer or a gynecologic malignancy frequently face a combined burden of endocrine disruption, treatment-related symptoms, psychological distress, fatigue, fear of recurrence, and conflicting dietary information. These factors may impair nutrition literacy, weaken confidence in dietary self-management, and reduce adherence to evidence-informed nutrition recommendations. This Mini Review examines the behavioral pathway linking psychological and educational barriers to dietary behaviors in women with breast and gynecologic malignancies. We focus on four interconnected domains: hormone-related emotional vulnerability and treatment-related symptom burden; anxiety, depression, fatigue, and fear of recurrence as drivers of avoidant or inconsistent eating behaviors; nutrition misconceptions and restrictive dietary beliefs that may increase malnutrition risk; and structured nutrition education as a self-management strategy. Existing guidelines support nutrition screening, individualized counseling, healthy dietary patterns, weight management, and physical activity as survivorship priorities, but implementation is often limited by low health literacy, fragmented communication, and insufficient psychosocial support. We propose that survivorship nutrition programs should integrate psychological screening, myth correction, plain-language nutrition literacy education, symptom-responsive meal planning, family involvement, and digital follow-up. Such behaviorally informed education should be evaluated using measurable outcomes, including dietary adherence, nutritional status, body weight and body composition, symptom control, quality of life, and sustained self-management across survivorship.

## 1 Introduction

Cancer survivorship among women is increasingly shaped by long-term physical, psychological, and behavioral needs rather than by tumor control alone. Breast cancer remains the largest female cancer survivor population worldwide, while gynecologic malignancies such as cervical, ovarian, endometrial, vulvar., and vaginal cancers also create sustained survivorship challenges related to treatment toxicity, endocrine disruption, fertility concerns, sexual health, fatigue, weight change, and psychosocial distress (1, 2). Nutrition has become a central component of this survivorship landscape because diet quality, body composition, physical activity, and metabolic status may influence treatment tolerance, comorbidity risk, quality of life, and cancer-related outcomes (3–10).

Guidance from the American Cancer Society, the European Society for Clinical Nutrition and Metabolism (ESPEN), and the World Cancer Research Fund (WCRF) emphasizes regular nutrition screening, prevention and treatment of malnutrition, healthy dietary patterns, avoidance of excess adiposity, and integration of dietary counseling with physical activity (3–6). However, the uptake of nutrition advice is strongly conditioned by patients’ psychological state and treatment context. Women with cancer often receive recommendations during periods of diagnostic shock, chemotherapy-related nausea, endocrine treatment symptoms, surgical recovery, social role disruption, and fear of recurrence. Under these conditions, dietary choices may be shaped as much by anxiety, fatigue, misinformation, family beliefs, and perceived control as by nutritional knowledge alone.

This Mini Review focuses on a specific behavioral pathway: hormone-related and treatment-related emotional vulnerability, psychological distress, anxiety/depression, fatigue, fear of recurrence, and dietary misconceptions may reduce nutrition literacy and dietary self-management, thereby affecting dietary adherence, body weight regulation, nutritional status, quality of life, and survivorship health. In this review, the term women’s cancers refers specifically to breast cancer and gynecologic malignancies (cervical, ovarian, endometrial, vulvar., and vaginal cancers), rather than all cancers diagnosed in women. Because the evidence base is most developed in breast cancer, breast-cancer-specific findings are identified explicitly and should not be assumed to generalize to every gynecologic malignancy. The aim is not to provide a comprehensive review of all nutrition interventions, but to define an educational and psychological framework for improving nutrition-oriented self-management in these survivorship populations.

## 2 Psychological and endocrine vulnerability in breast and gynecologic cancer survivorship

Women with cancer may experience psychological vulnerability from the disease itself, but also from treatment-induced changes in the endocrine and neurobehavioral environment. Breast cancer therapies can include chemotherapy-induced ovarian insufficiency, ovarian suppression, tamoxifen, aromatase inhibitors, and premature or intensified menopausal symptoms. Gynecologic cancer treatment may involve oophorectomy, pelvic radiotherapy, chemotherapy, or abrupt loss of reproductive and hormonal function. These endocrine and symptom changes can interact with mood, sleep, appetite, body image, sexual function, and perceived identity. Although the mechanisms differ across cancer types and treatments, the clinical consequence is similar: many women must make daily dietary decisions while coping with physical symptoms and emotional instability. Evidence concerning endocrine symptoms and psychological burden is uneven across these malignancies; findings from breast cancer cohorts should therefore be extrapolated to gynecologic cancer survivors only with appropriate caution.

Psychological distress is common across cancer survivorship. Systematic evidence shows increased anxiety, depression, sleep disturbance, and other mental health outcomes among breast cancer survivors compared with women without cancer (11, 12). Depression and anxiety may also have prognostic relevance in breast cancer, although causal pathways are difficult to separate from treatment burden, comorbidity, social support, and health behavior patterns (13, 14). Distress can disrupt appetite, meal planning, motivation, social eating, and the cognitive capacity required to interpret nutrition advice. Some women respond to distress through restrictive eating and avoidance, while others rely on high-sugar or energy-dense foods for comfort, reward, or fatigue management. Both patterns can undermine balanced survivorship nutrition.

The behavioral consequences are clinically important. Cancer nutrition requires repeated self-regulatory actions: understanding dietary advice, selecting safe and appropriate foods, managing nausea or taste changes, maintaining protein intake, adjusting meals around fatigue, monitoring weight, and avoiding unverified dietary claims. Psychological distress can reduce executive function and self-efficacy, thereby weakening the translation of knowledge into behavior. This suggests that nutrition education for women with cancer should not be delivered as isolated information. It should be embedded within supportive communication that recognizes emotional burden, endocrine symptoms, family context, and the patient’s perceived ability to act.

## 3 Fear of recurrence, fatigue, and dietary self-regulation

Fear of cancer recurrence is one of the most persistent psychological concerns in cancer survivorship. It is defined as worry or concern that cancer will return or progress, and it can range from an adaptive response to a clinically significant source of impairment (15, 16). In women with breast cancer, fear of recurrence has been associated with distress, health information seeking, reassurance-seeking, avoidance, and altered health behaviors (17, 18). A moderate level of concern may motivate protective behaviors, but high fear can produce rigid, fear-driven, or inconsistent dietary practices.

The relationship between fear and nutrition behavior is not linear. Some survivors interpret dietary control as a way to regain agency after cancer treatment. This can support healthier eating when guidance is evidence-based and feasible. However, fear can also intensify vulnerability to misinformation, especially claims that sugar, dairy, soy, acidic foods, or particular food categories directly “feed” cancer or guarantee recurrence prevention. Such claims may lead to unnecessary restrictions, social eating anxiety, nutritional inadequacy, and conflict with family dietary practices. Recent evidence indicates that exposure to cancer misinformation is common among breast cancer survivors, reinforcing the need for structured and credible survivorship communication (19).

Cancer-related fatigue further complicates dietary self-regulation. Fatigue is frequent after breast cancer treatment and may persist for years (20, 21). It reduces motivation to shop, cook, plan meals, and engage in physical activity. Fatigue may also reinforce passive dietary patterns, reliance on convenience foods, irregular meals, or insufficient protein intake. Nutritional interventions targeting fatigue suggest that whole-food, plant-forward, and anti-inflammatory dietary patterns may be feasible and may improve fatigue-related symptoms in some survivors, although evidence remains heterogeneous (22, 23). For this reason, dietary education should include practical strategies that match fatigue severity: simple protein-rich meals, batch cooking, safe convenience foods, family-assisted meal preparation, and symptom-responsive eating plans. For example, low-burden plans can combine ready-to-eat high-protein snacks such as yogurt, milk or fortified soy beverages, eggs, or nut butter with batch-cooked meals, prewashed produce, and caregiver-assisted shopping or preparation, when tolerated and consistent with food-safety guidance.

A useful framework is to view fear of recurrence and fatigue as self-regulation modifiers. They do not simply create “poor adherence”; they alter how patients perceive risk, weigh trade-offs, seek information, and convert recommendations into daily choices. Effective nutrition education should therefore include cognitive reassurance, emotional validation, practical behavioral planning, and clear boundaries between evidence-based dietary advice and unverified restrictive claims.

## 4 Nutrition literacy and dietary self-management

Nutrition literacy is more than knowing which foods are healthy. It refers to the ability to obtain, understand, evaluate, and apply nutrition information in real-life decisions (24, 25). In cancer care, this includes understanding treatment-related nutrition needs, identifying credible sources, interpreting food safety and supplementation advice, managing symptoms that affect intake, and adapting recommendations to budget, culture, family roles, and personal preference.

Breast cancer studies show that nutrition literacy and dietary attitudes are associated with dietary behavior among patients receiving chemotherapy (25). Measurement work has also adapted nutrition literacy instruments specifically for breast cancer populations, highlighting that general health literacy tools may not capture oncology-specific dietary concepts such as phytoestrogens, supplement interactions, weight change during therapy, and cancer-related dietary myths (24). More recent studies have developed and evaluated dietary self-management behavior instruments for breast cancer patients during chemotherapy, reflecting growing recognition that dietary behavior is an active self-management process rather than passive adherence (26).

Dietary self-management includes information seeking, meal planning, symptom management, food selection, supplement decisions, communication with clinicians, and monitoring of body weight or gastrointestinal symptoms. It depends on self-efficacy and social support. Women with higher education or stronger family support may access and apply nutrition guidance more effectively, whereas women with low literacy, limited access to dietitians, cultural food taboos, or conflicting online information may experience greater difficulty.

A key educational challenge is that many women with cancer are highly motivated to improve their diets, but motivation alone is insufficient. Without structured guidance, motivation can turn into confusion or overrestriction. In some cultural contexts, patients may avoid specific foods believed to provoke relapse, inflammation, or poor wound healing, despite limited evidence. Others may use supplements, fasting, alkaline diets, ketogenic diets, or other restrictive regimens without adequate clinical monitoring. Thus, nutrition literacy programs should be designed as practical decision-support interventions. They should teach patients how to evaluate claims, ask clinicians targeted questions, recognize red flags of misinformation, and choose dietary strategies that are nutritionally adequate, psychologically sustainable, and clinically safe.

## 5 From dietary misconceptions to nutritional vulnerability

Dietary misconceptions are not trivial in oncology. They can directly change eating behavior, produce guilt or fear around food, and delay appropriate nutrition support. Common misconceptions include the belief that sugar must be completely eliminated, that all soy or dairy is unsafe after breast cancer, that fasting or ketogenic diets are universally anticancer, that “superfoods” can replace treatment, or that food taboos can prevent recurrence. Reviews of anticancer restrictive diets caution that clinical benefits remain uncertain in many settings and that unsupervised restriction may increase risks of malnutrition, micronutrient deficiency, social burden, and psychological distress (27, 28).

For women undergoing treatment, the consequences may be immediate. Chemotherapy, surgery, radiotherapy, endocrine therapy, and targeted therapy can cause nausea, mucositis, dysgeusia, anorexia, diarrhea, constipation, pain, sleep disturbance, and fatigue. If these symptoms occur alongside fear-driven food avoidance, energy and protein intake may fall below requirements. Conversely, emotional eating and reduced activity may contribute to weight gain, particularly in breast and endometrial cancer survivorship. Both undernutrition and excess adiposity can impair survivorship health.

Body weight regulation is especially relevant to women’s cancers. Obesity has been associated with worse prognosis in breast cancer, and excess adiposity can influence estrogen production, insulin resistance, inflammatory signaling, and cardiometabolic comorbidity (29, 30). At the same time, weight-focused counseling must be handled carefully. Women with cancer may already experience body image disturbance, menopausal symptoms, lymphedema, surgical changes, or distress around weight gain. A purely weight-centric message can intensify shame and reduce engagement. A more appropriate educational approach emphasizes strength, function, metabolic health, dietary adequacy, and sustainable behavior change. Accordingly, weight-management goals should prioritize preservation of muscle, metabolic health, physical function, and treatment tolerance rather than appearance-based targets or personal blame.

Nutritional vulnerability also extends to body composition. Loss of muscle mass and poor muscle quality may affect treatment tolerance, fatigue, function, and survival, even when body weight appears normal or elevated (31–33). Therefore, dietary education should avoid simple messages such as “eat less” or “avoid carbohydrates.” Instead, it should address protein adequacy, resistance activity when feasible, symptom-responsive food texture, dietary fiber, micronutrient sufficiency, and safe use of oral nutrition supplements when clinically indicated. Because much of the body-composition evidence is derived from mixed or advanced-cancer cohorts, cancer-specific application requires cautious clinical interpretation.

## 6 Structured nutrition education and self-management support

A behaviorally informed education model should integrate nutrition science, psychology, and education management. First, education should begin with assessment. Clinicians should assess nutrition risk, weight trajectory, dietary intake, symptoms affecting food consumption, psychological distress, fatigue, fear of recurrence, health literacy, access to food, family support, and use of supplements or restrictive diets. This avoids the common problem of giving generic advice to patients with different barriers.

Second, education should be modular and plain-language. Core modules may include: basic survivorship dietary pattern; protein and energy needs during treatment; safe weight management; symptom-responsive eating; food safety; supplement caution; misinformation recognition; and when to seek dietitian referral. Educational materials should be short, culturally adapted, and repeated over time because one-time counseling is unlikely to change behavior under distress. Visual tools, meal examples, and practical scripts for asking clinicians about diet can improve usability. These principles are consistent with survivorship, lifestyle-intervention, and health-literacy frameworks that emphasize practical, repeated, and literacy-sensitive support (34–39).

Third, psychological barriers should be addressed explicitly. For patients with high fear of recurrence, educators can distinguish between controllable health behaviors and unrealistic dietary certainty. For patients with anxiety or depression, dietary goals should be small, achievable, and linked to self-efficacy rather than perfection. For patients with fatigue, plans should reduce cognitive and physical burden through meal simplification, family assistance, and planned use of safe prepared foods. For patients exposed to misinformation, clinicians should correct myths without dismissing the patient’s need for control and hope.

Fourth, multidisciplinary delivery is preferable. A pilot multidisciplinary intervention in women receiving chemotherapy for breast cancer suggests that structured diet and lifestyle education can improve dietary management behavior, self-care self-efficacy, and quality of life (40). Nurses, dietitians, oncologists, psychologists, social workers, and trained community health workers each contribute different competencies. In resource-limited settings, stepped-care models may be useful: brief screening and standardized education for all patients, targeted dietitian referral for high-risk patients, and psychological referral for those with clinically significant distress or fear.

Fifth, digital and family-supported approaches can extend reach. Technology-supported nutrition and physical activity interventions may provide flexible access for cancer survivors, though quality and personalization vary (41, 42). For women with family caregiving responsibilities, family involvement may improve grocery choices, meal preparation, and adherence to evidence-based dietary recommendations. However, family members may also transmit restrictive beliefs. Education should therefore include caregivers when possible and provide consistent messages for the household.

**Figure 1:**
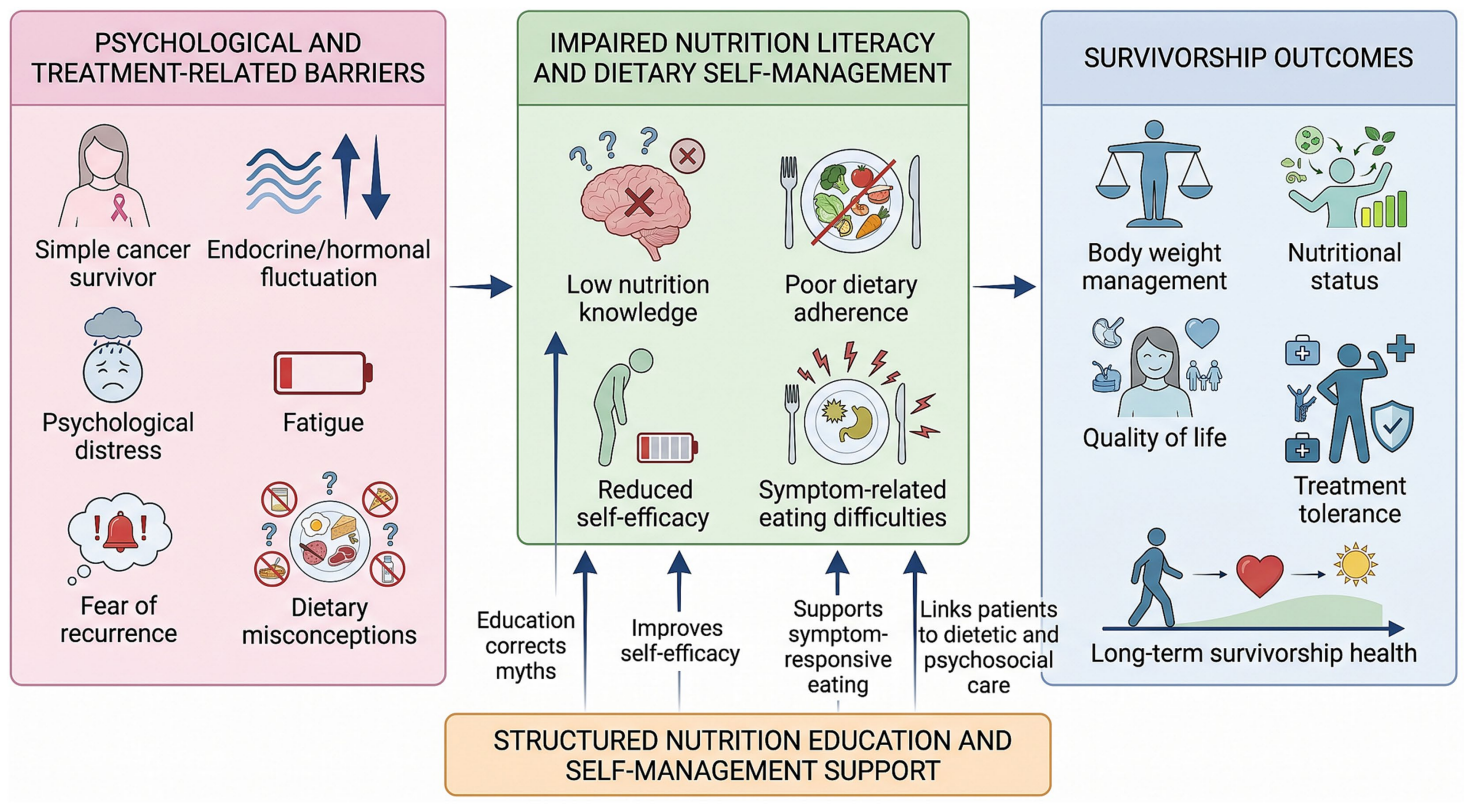
Behavioral pathway linking psychological barriers to nutrition literacy and dietary self-management in breast and gynecologic cancer survivorship. Treatment-related symptoms, endocrine disruption, psychological distress, fatigue, fear of recurrence, and dietary misconceptions may impair nutrition literacy and self-management. Structured education can intervene by correcting myths, improving self-efficacy, supporting symptom-responsive eating, and linking patients to dietetic and psychosocial care.

The goal is not to make patients follow rigid rules, but to build dietary self-management capacity. Effective programs should help women understand credible nutrition guidance, adapt it to symptoms and culture, maintain dietary adequacy, avoid harmful restrictions, regulate weight safely, and seek help when problems arise. This aligns survivorship nutrition with public health principles: literacy, empowerment, equity, and sustainable behavior change. Figure 1 summarizes the proposed behavioral pathway, and Table 1 links major barriers to their behavioral consequences, nutrition risks, and educational priorities.

**Table 1:** Psychological and educational barriers to nutrition self-management after breast and gynecologic cancers and corresponding intervention priorities.

Barrier	Behavioral consequence	Nutrition risk	Educational priority
Anxiety and depressive symptoms	Reduced motivation, irregular meals, emotional eating, avoidance of clinic communication	Poor diet quality, inadequate intake, excess energy intake in some patients	Brief screening, achievable dietary goals, plain-language counseling, psychological referral when indicated
Cancer-related fatigue	Difficulty shopping, cooking, meal planning, and maintaining routine	Insufficient protein and energy intake or reliance on convenience foods	Low-effort meal plans, caregiver support, protein-rich ready-to-use options, symptom-responsive strategies
Fear of recurrence	Rigid food rules, reassurance-seeking, overinterpretation of dietary risk	Unnecessary restriction, guilt around eating, unstable adherence	Differentiate evidence-based risk reduction from false certainty; support flexible control
Dietary misinformation	Use of unverified restrictive diets, supplements, or online advice	Micronutrient deficiency, treatment interaction, financial burden	Myth correction, credible source lists, warning signs for misinformation, clinician-patient dialogue
Low nutrition literacy	Difficulty understanding food labels, protein/calorie needs, supplement advice, and treatment-related diet guidance	Delayed nutrition support and poor self-management	Teach-back method, visual materials, culturally adapted examples, repeated education
Family and cultural food beliefs	Food taboos or caregiver-driven overfeeding/restriction	Conflict, distress, inadequate or excessive intake	Include caregivers, clarify cultural beliefs respectfully, create household-level dietary plans

## 7 Conclusion

Nutrition literacy and dietary self-management remain underdeveloped components of survivorship after breast and gynecologic cancers. Psychological distress, endocrine and treatment-related symptoms, fatigue, fear of recurrence, and dietary misinformation can disrupt dietary adherence, weight regulation, nutritional status, symptom control, and quality of life. These barriers reflect the emotional, cognitive, social, and informational demands of living with cancer rather than a simple lack of motivation.

Survivorship nutrition care should therefore integrate brief psychological and nutrition-risk screening, plain-language nutrition literacy education, myth correction, symptom-responsive dietary planning, caregiver support, and clear referral pathways to dietetic and psychosocial services. Future studies should evaluate dietary adherence, symptom control, nutritional status, body composition, quality of life, and sustained self-management across cancer types and survivorship phases.
